# Targeting phosphorylation of STAT3 delays tumor growth in HPV-negative anal squamous cell carcinoma mouse model

**DOI:** 10.1038/s41598-017-06643-9

**Published:** 2017-07-26

**Authors:** Lin-Lin Bu, Yi-Cun Li, Guang-Tao Yu, Jian-Feng Liu, Wei-Wei Deng, Wen-Feng Zhang, Lu Zhang, Zhi-Jun Sun

**Affiliations:** 1 The State Key Laboratory Breeding Base of Basic Science of Stomatology & Key Laboratory of Oral Biomedicine Ministry of Education, Wuhan University, Wuhan, 430079 China; 2 Department of Oral and Maxillofacial-Head and Neck Oncology, School and Hospital of Stomatology, Wuhan University, Wuhan, 430079 China

## Abstract

Although conventional chemoradiotherapy is effective for most anal squamous cell carcinoma (ASCC) patients, HPV-negative ASCC patients respond poorly to this treatment and new therapeutic approach is required. Our group has previously established an HPV-negative ASCC mouse model and demonstrated that signal transducer and activation of transcription 3 (STAT3) is hyper-activated in the model. Here, we show that *in vivo* inhibition of STAT3 by S3I-201 effectively delays tumor growth in ASCC mouse model indicated by significantly smaller tumor size and burden in the treatment group compared with control group at the same point. Further analysis shows that survivin and Ki67, important biomarkers for tumor cell survival and proliferation, are significantly reduced after S3I-201 treatment. Additionally, flow cytometry and immunohistofluorescent assays reveal decreased Myeloid-derived suppressor cell (MDSC) and tumor-associated macrophage (TAM) populations in the S3I-201 treatment group, which indicates a reversion of the immunosuppressive environment, unraveling the potential role for S3I-201 in immunosuppression in ASCC. Together these results for the first time demonstrated the anti-tumor effects of STAT3 inhibitor S3I-201 in HPV-negative ASCC mouse model and its multiple effects on cancer cells and immune system. Thus we conclude that S3I-201 may be a novel therapeutic approach for HPV-negative ASCC patients.

## Introduction

Anal cancer refers to malignant tumors that arise from anal canal or anal margin^1^. It is an uncommon malignancy, accounting for only 0.43% of all cancers and 2.5% of all gastrointestinal (GI) cancers^2^. Nevertheless, the incidence of this disease has been remarkably increasing in the United States in the past several decades: 2.5-fold in men and 5-fold in women^3, 4^, rendering it an issue of concern for medical researchers. Old age, race, sexual activity, HPV infection, immunosuppression, and smoking are among the risk factors for anal cancer. 85% of anal cancers are pathologically diagnosed as anal squamous cell carcinoma (ASCC)^2, 5^. Chemoradiotherapy is currently the standard therapy for ASCC, but the poor response of HPV-negative ASCC patients and toxic reaction to this treatment have limited its use and warranted new therapeutic approach^6–8^. According to a recent report, HPV-negative ASCC cases also have a shorter median survival time than HPV-positive cases^9^.

The molecular mechanisms of ASCC are little known and there is limited research in this area. Signal transducer and activation of transcription 3 (STAT3) is a member of latent cytosolic transcription factors, which transduces the signal from epidermal growth factor (EGF) or interleukin-6 (IL-6) and act as a transcriptional factor^10^. In a previous experiment^11^, we have introduced a transgenic mouse model that could spontaneously develop anal squamous cell carcinoma without using carcinogens like dimethylbenzanthracene (DMBA) or 12-O-tetradecanoylphorbol-13-acetate (TPA). In mice with ASCC, we had observed that phosphorylated STAT3 (p-STAT3) was highly activated compared with that in control group^11^. Activated STAT3 also up-regulates *BIRC5*, a highly-expressed gene in many cancers and whose product (survivin) inhibits apoptosis of cancer cells^12^. In light of these observations, and considering that STAT3 is a crucial part in the regulation of physiology of cancer cells and immune cells^11, 13–20^, we hypothesized that an STAT3 inhibitor would be able to exert a curative effect on mice with ASCC.

To test these hypotheses, we examined the effects of S3I-201, a selective inhibitor of the activation and DNA-binding activity of STAT3 signaling, in HPV-negative ASCC mouse model.

## Results

### The increased expression of p-STAT3 and expanded population of MDSC and TAM in mice with ASCC

**Figure 1:**
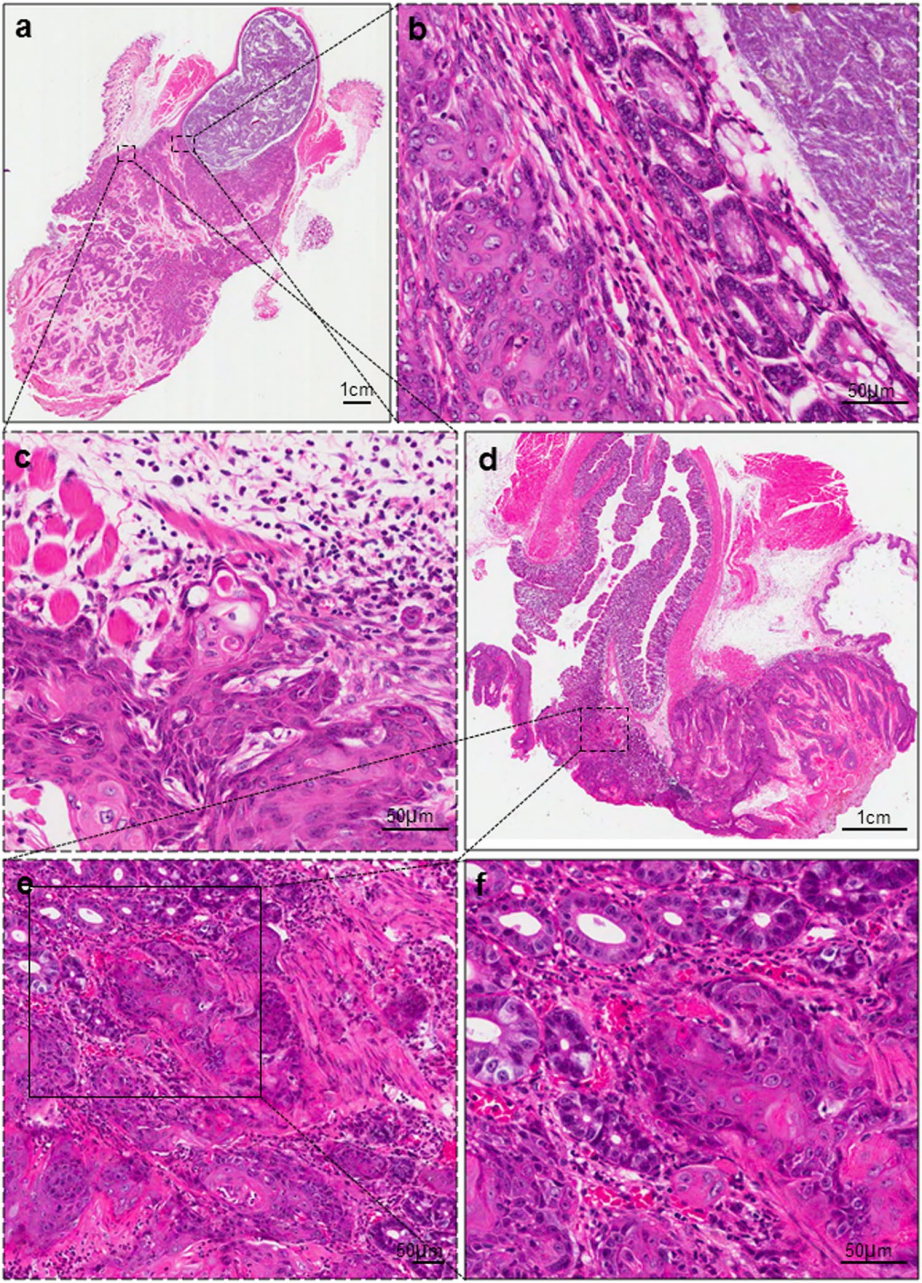
Histological views of ASCC in *Tgfbr1/Pten* 2cKO mice. (**a**) A representative sagittal section of mice ASCC. Scale bar = 1 cm (**b**,**c**) High-power fields of the tumor margins. Tumor islands were extended into underlying tissue with well-defined invasive front and accompanied by stromal reaction. Distinct nuclear pleomorphism was seen. Scale bar = 50 μm. (**d**) Another representative sagittal section of mice ASCC. Scale bar = 1 cm (**e**,**f**) High-power fields of the tumors showing tumor cords invaded into the muscle layer. Nebulous infiltrating margins and discontinuous basal layers were seen. Cancer cells were forming irregular small strands or cords with poor keratinization. Densely infiltrating lymphocytes were seen. Scale bar = 50 μm.

**Figure 2:**
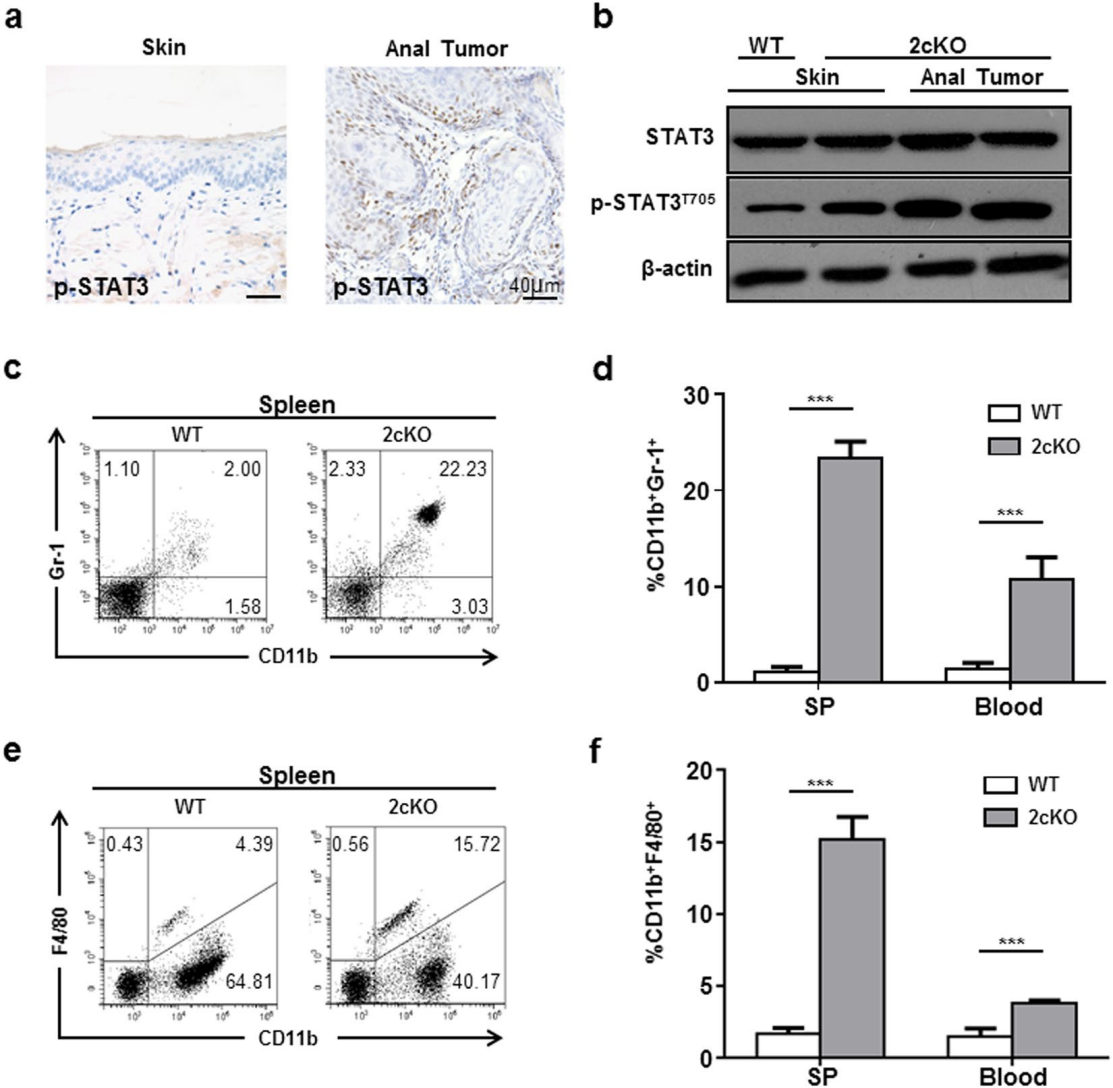
MDSC and TAM populations were expanded in mice with ASCC. (**a**) Immunohistochemical staining of the perianal skin and anal tumor showed that p-STAT3 was upregulated in both cancer cells and infiltrating immune cells in anal tumor (**b**) Western blot showed the phosphorylation level of STAT3 was upregulated in mice with the anal tumor. (**c**,**d**) CD11b^+^Gr-1^+^ MDSC population was expanded in spleen and blood of mice with ASCC. (**e**,**f**) CD11b^+^F4/80^+^ TAM population was expanded in spleen and blood of mice with ASCC. ***P < 0.001.

Our previous work reported a spontaneous development of ASCC in a mice model by tamoxifen-inducible deletions of important tumor suppressors *Pten* and *Tgfbr1* ^11^. 3 weeks after the start of tamoxifen induction, *Tgfbr1/Pten* 2cKO mice began to develop anal cancer. Histological features of anal cancer were examined at different magnifications (Fig. 1a–f). p-STAT3 was upregulated in both cancer cells and infiltrating immune cells shown in immunohistochemical staining of perianal skin and anal tumor (Fig. 2a). Lysates of anal tumors from tumor-bearing mice showed significantly higher expression of p-STAT3 compared to perianal skins from wild type (WT) mice (Fig. 2b). To test the changes of immune environment in mice with ASCC, we detected MDSC and TAM populations in tumor-bearing and WT mice. The results showed significantly expanded CD11b^+^Gr-1^+^ MDSC population (Fig. 2c and d) and CD11b^+^F4/80^+^ TAM population (Fig. 2e and f) in the tumor-bearing mice.

### Inhibition of STAT3 by S3I-201 decreases the tumor burden in *Tgfbr1/Pten* 2cKO mice

**Figure 3:**
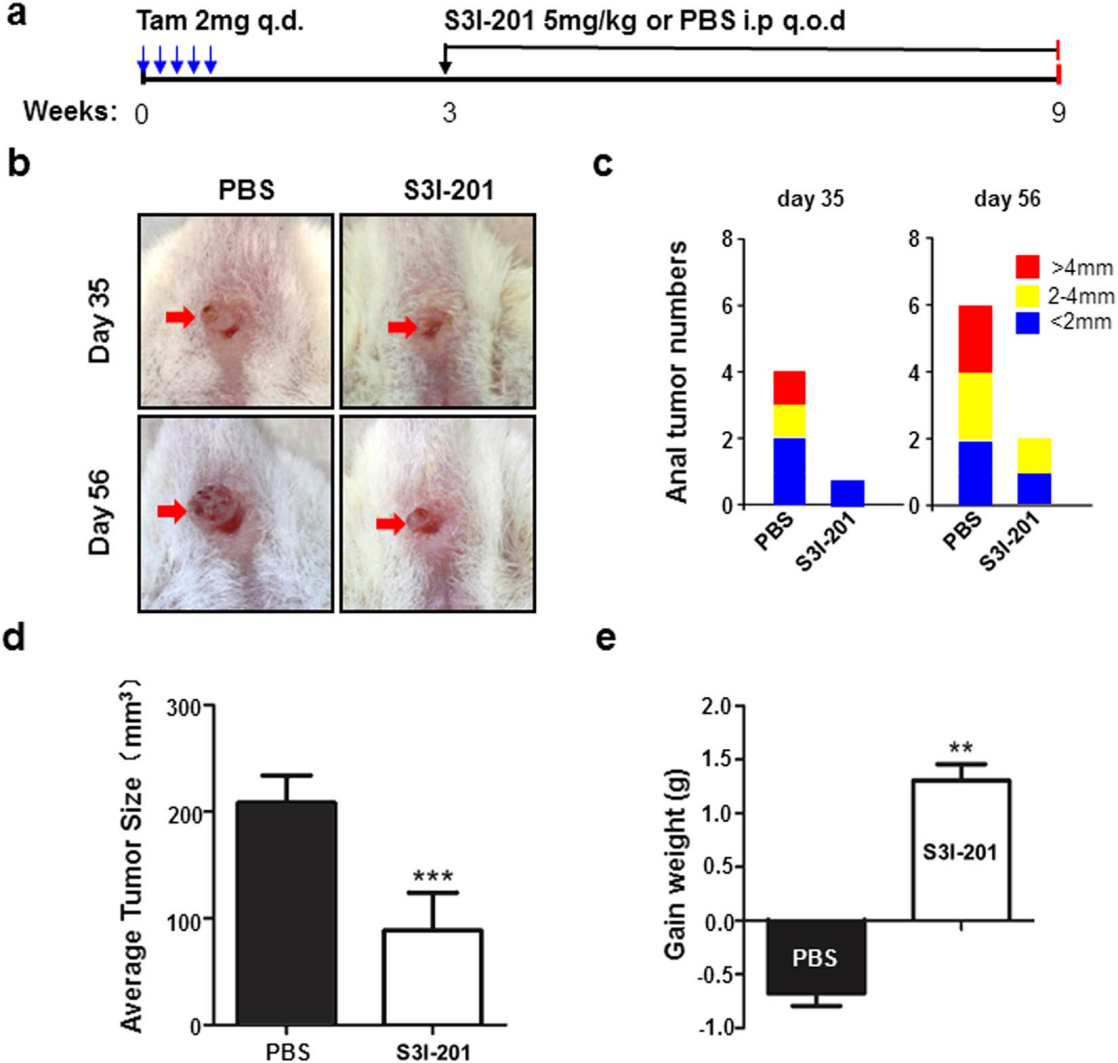
S3I-201 treatment protected mice from tumor challenge. (**a**) A schematic flow chart showed the drug delivery strategy of S3I-201 in the chemopreventive tumorigenesis experiment in *Tgfbr1/Pten* 2cKO mice. (**b**) Pictures of visible tumors were photographed at day 35 and day 56 in control group and S3I-201 chemopreventive group. The number of mice with ASCC (**c**) or the average sizes of tumor (**d**) were significantly reduced after S3I-201 treatment. And mice in the S3I-201 treatment group did not suffer from extra body weight loss (**e**), which is an indicator of drug toxicity. **P < 0.01; ***P < 0.001.

Based on our previous experiment^11^, STAT3 was activated in *Tgfbr1/Pten* 2cKO mouse ASCC. Considering that STAT3 was not commonly activated in normal epithelial cells, it would be interesting to investigate whether the inhibition of STAT3 signaling could decrease tumor progression in mouse ASCC. Mice were randomly divided into the control group or the experimental group. PBS or STAT3 inhibitor (S3I-201) was injected i.p. according to the drug delivery strategy shown in Fig. 3a. Representative pictures of anal tumors in control and experimental group at day 35 and day 56 were shown (Fig. 3b). The incidence rate was evidently lower in the S3I-201 treatment group (40% vs 10% at day 35, 60% vs 20% at day 56, Fig. 3c). And the tumor burden in control group was much higher than that in the S3I-201 treatment group (Fig. 3d). In addition, as an indicator of drug toxicity, mice in the S3I-201 treatment group suffered significantly less body weight loss than mice in the control group (Fig. 3e).

### Inhibition of STAT3 down-regulates survivin and Ki-67 in *Tgfbr1/Pten* 2cKO mice

**Figure 4:**
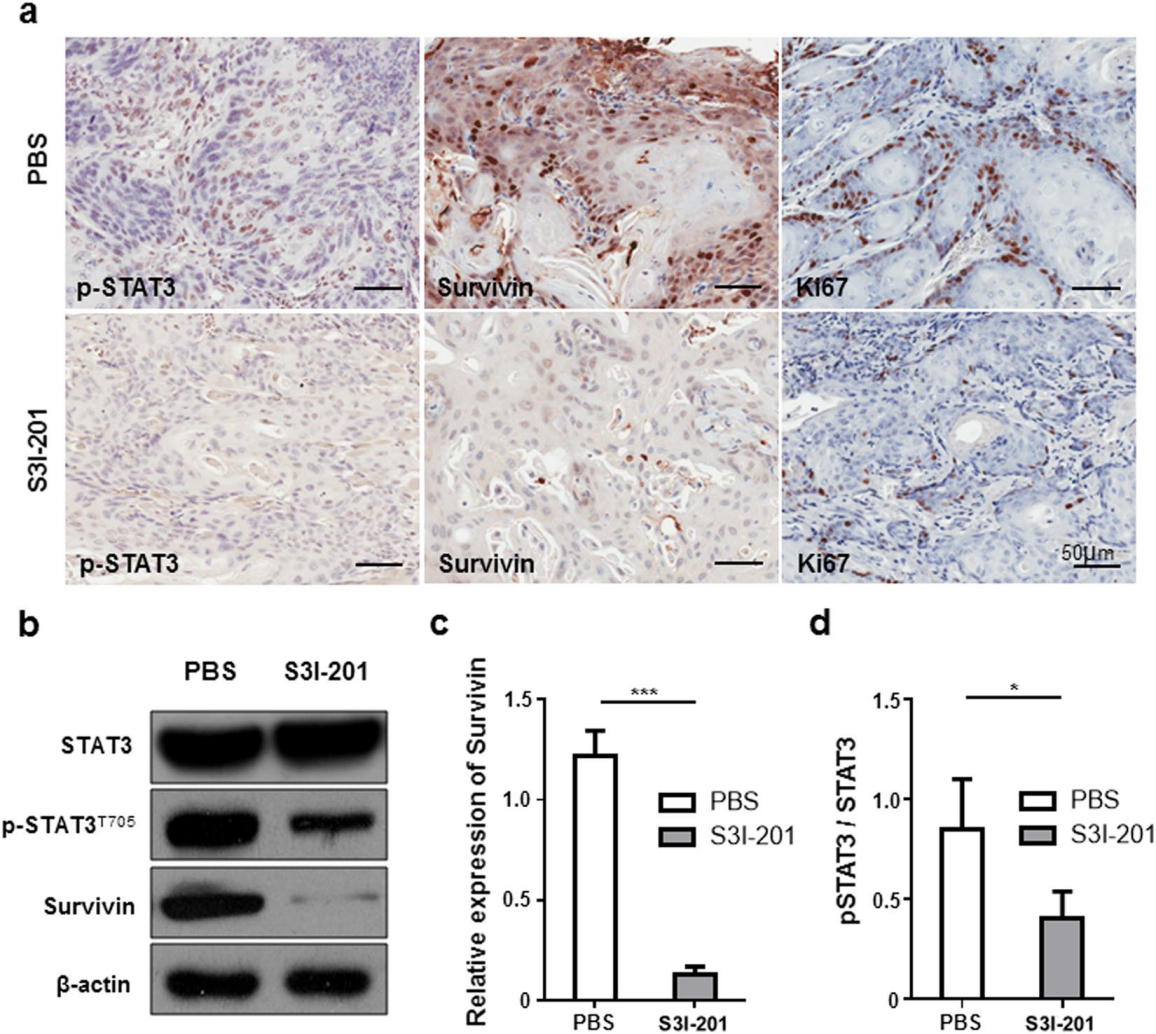
Inhibition of STAT3 reduced the expression of survivin and Ki-67 and induced apoptosis. (**a**) Representative views of the expression of p-STAT3, survivin and Ki-67 in S3I-201 treatment group and control group. (**b**) The expression levels of p-STAT3 and survivin were remarkably reduced after S3I-201 treatment,and their relative quantifications (**c**,**d**). *P < 0.05; ***P < 0.001.

To gain further insights into the effects of STAT3 inhibitor S3I-201 on tumor cells, we analyzed the expression level of p-STAT3, survival maker survivin and proliferation marker Ki-67 in ASCC control and S3I-201 groups. The phosphorylation of STAT3 has been significantly blocked after the injection of S3I-201 (Fig. 4a,b and d). The inhibition of STAT3 significantly reduced the expression of survivin compared with those in control group (Fig. 4a,b and c). As a proliferation index, Ki-67 was also notably reduced after STAT3 inhibition (Fig. 4a). These results indicated that S3I-201 reduced the expression of survivin which induced increased apoptosis and decreased proliferation through STAT3 inhibition.

### Inhibition of STAT3 decreases MDSC and TAM population while increases CD4^+^ and CD8^+^ population in *Tgfbr1/Pten* 2cKO mice to enhance anti-tumor responses

**Figure 5:**
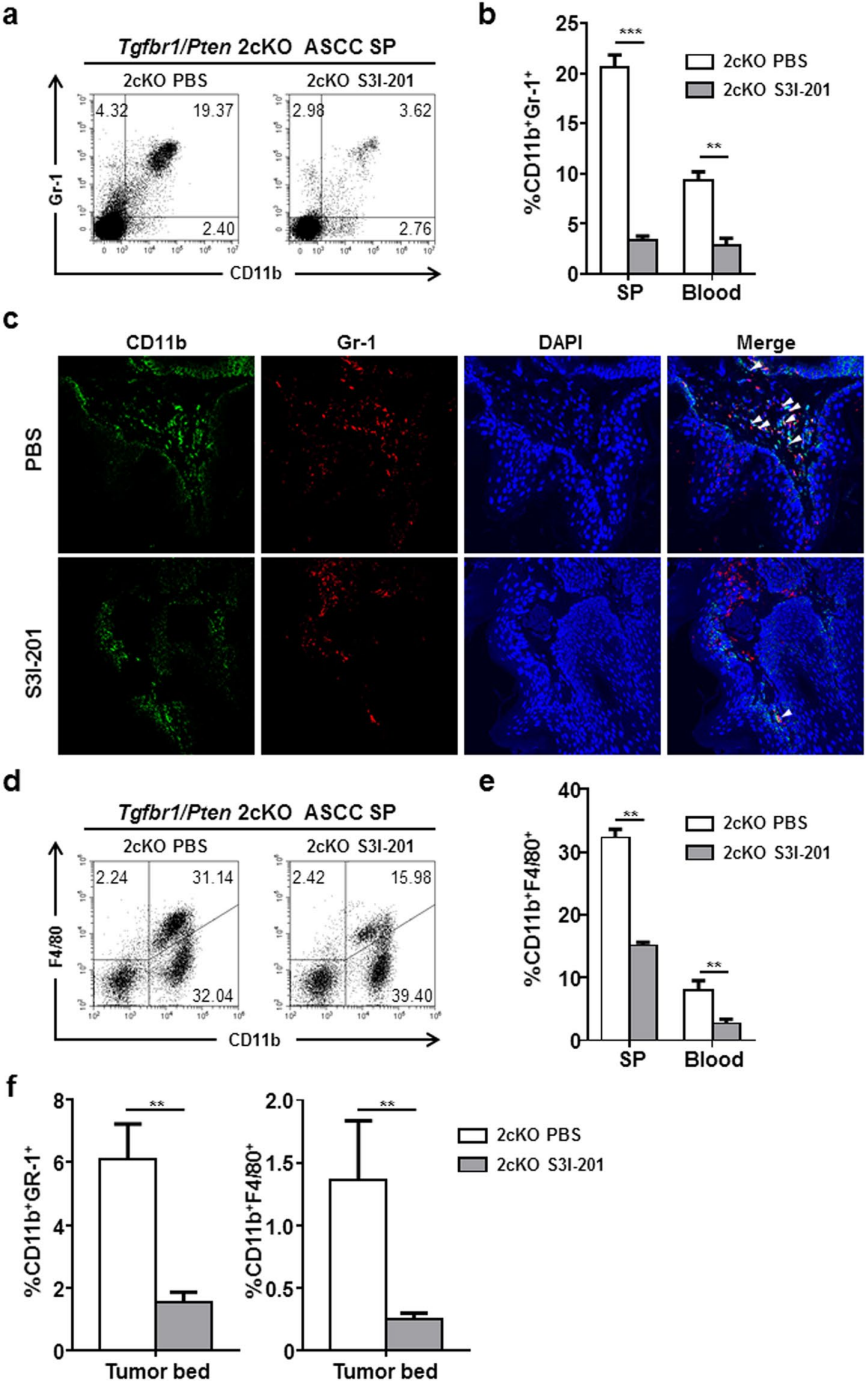
Inhibition of STAT3 reduced MDSC and TAM population in mice with ASCC. (**a**) MDSC population was monitored by CD11b^+^Gr-1^+^flow cytometry in peripheral blood and spleen. S3I-201 treatment notably reduced the proportion of MDSCs in total lymphocytes. (**b**) Quantification of the results. (**c**) Representative immunofluorescent staining of CD11b^+^Gr-1^+^ in mice ASCC. Visible reduction of MDSCs populations in the S3I-201 treatment group was seen. (**d**) TAM population was monitored by CD11b^+^F4/80^+^ flow cytometry in peripheral blood and spleen. S3I-201 treatment notably reduced the proportion of TAMs in total lymphocytes. (**e**) Quantification of the results. (**f**) CD11b^+^Gr-1^+^ and CD11b^+^F4/80^+^ populations in the tumor bed were significantly reduced after S3I-201 treatment. **P < 0.01; ***P < 0.001.

To study the effects of S3I-201 on immunosuppressive cells, we then analyzed the immune status in mice by measuring the proportion of MDSC in total immune cells using flow cytometry. Data showed that S3I-201 treatment significantly lessened the proportion of MDSC compared with that in control group (Fig. 5a and b). This result was visually confirmed by immunofluorescence in the tissue samples of mice in each group (Fig. 5c). S3I-201 treatment also significantly reduced TAM population compared to control group (Fig. 5d and e). Then, to measure the impact of S3I-201 on macrophage and myeloid cell compartment of the tumor bed, we analyzed the proportion of MDSCs and TAMs in tumor infiltrating immune cells. Data showed that MDSCs and TAMs population were significantly reduced after S3I-201 treatment (Fig. 5f and Fig. S1a).

**Figure 6:**
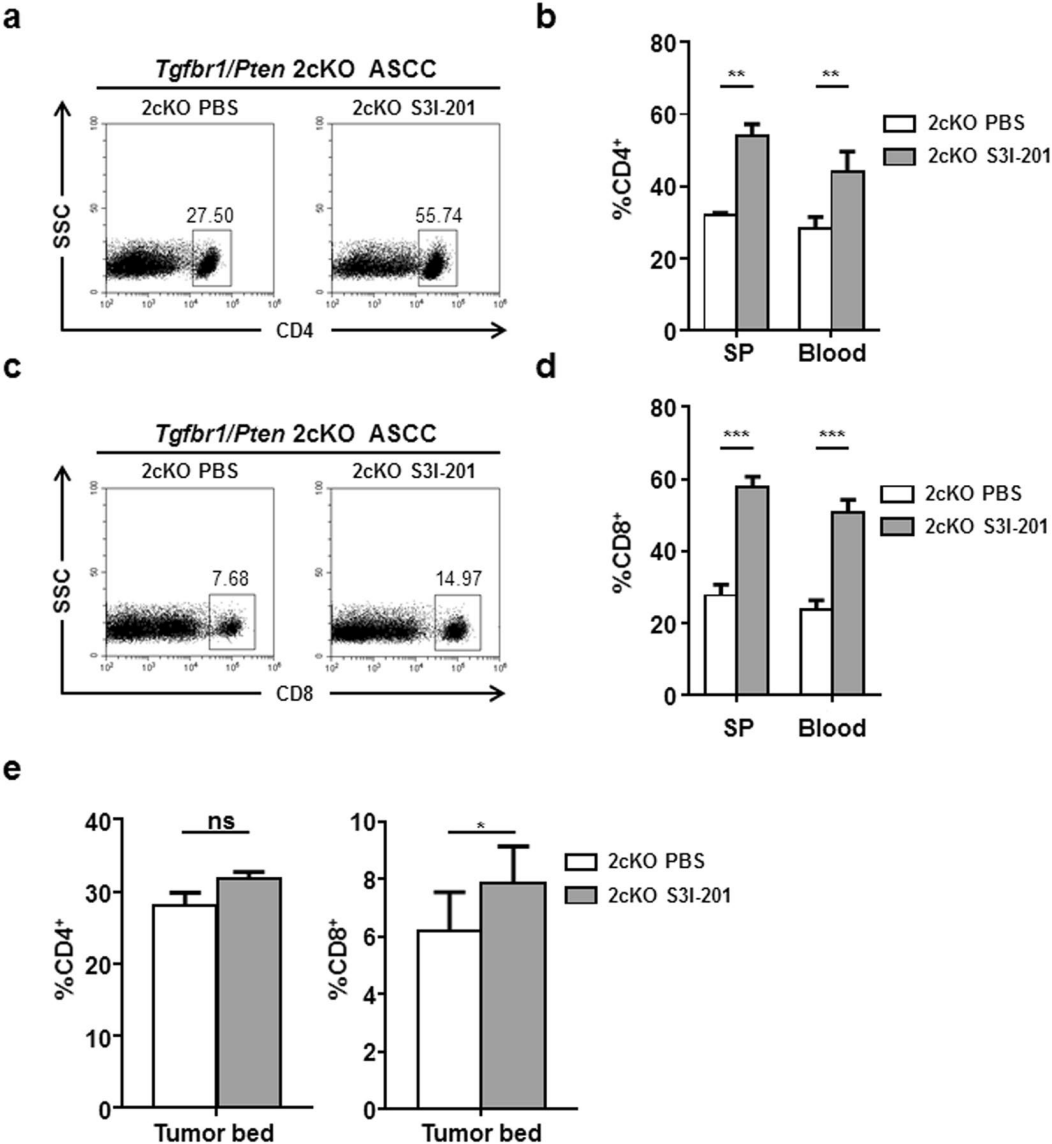
Inhibition of STAT3 increased CD4^+^ and CD8^+^ T cells in mice with ASCC. (**a**,**b**) CD4^+^ population was expanded after S3I-201 treatment. (**c**,**d**) CD8^+^ population was expanded after S3I-201 treatment. (**e**) Quantification of CD4^+^ and CD8^+^ T cells in the tumor bed. **P < 0.01; ***P < 0.001.

The numbers CD4+ and CD8+ T cells are also critical to anti-tumor immunity. We analyzed the numbers of CD4+ and CD8+ T cells in the spleen and blood of *Tgfbr1/Pten* 2cKO mice by flow cytometry. In spleen, blood, S3I-201 treatment significantly increased the number of CD4+ (Fig. 6a and b) and CD8+ (Fig. 6c and d) T cells. The number of CD4+ T cells in the tumor bed was not significantly changed while the number of CD8+ T cells in the tumor bed was significantly increased (Fig. 6e and Fig. S1a). These results suggested a recovery of anti-tumor immunity after S3I-201 treatment.

## Discussion

Although conventional chemoradiotherapies have achieved a relatively high complete response rate, there is still an urgent need to develop new therapies, considering the existence of HPV-negative patients who respond poorly to conventional treatment^8^. One representative of these new biologic therapies is cetuximab. As an EGFR inhibitor, cetuximab received promising results in some case reports when used alone or in combination with other chemotherapies^21, 22^. However, recent studies show that cetuximab combined with conventional chemotherapy may have serious adverse effects on patients with locally advanced anal cancer^23, 24^. Here we reported that STAT3 inhibitor S3I-201 had a considerable curative effect on HPV-negative ASCC mice without appreciable toxic reaction, in contrast with cetuximab. Besides, S3I-201 may achieve a better therapeutic effect when used in combination with chemotherapy due to its potential of reversing chemo-resistance as a STAT3 inhibitor.

Our data suggested that S3I-201 works both-ways to suppress cancer cells. Firstly, inhibiting STAT3 activation in cancer cells hampered tumor cells survival and proliferation. In a previous study we reported that STAT3 has been activated in the anal tumor^11^. Activated STAT3 in turn promotes tumor growth through STAT3-responsive genes. However, the specific role of STAT3 in tumorigenesis of human anal cancer is still poorly understood and warrant further investigation. Accumulating evidence also indicates a pivotal role of STAT3 in the chemotherapy resistance in several human cancers, such as breast cancer, ovarian cancer and gastric cancer^16, 18, 19^. Furthermore, the reversion of chemo-resistance was observed after STAT3 signaling had been blocked^18, 19^. Survivin also contributes to chemo-resistance in cancer patients, and its transcription was induced by the activation of STAT3^14, 15, 20^. These raised a question about whether the combination of STAT3 inhibitor and chemotherapy would have greater anti-tumor effects in ASCC mouse model. Further study of such combined therapy remains to be done.

Secondly, inhibiting STAT3 activation reduces the population of immunosuppressive cells. It has been known that STAT3 signaling has a regulatory function in immunocytes. Two recent animal experiments have shown that STAT3 functioned as a key regulator of the activity and expansion of myeloid-derived suppressor cells (MDSCs). When STAT3 was activated, MDSCs promoted tumor growth by suppressing T cells and expanding Cancer stem cells (CSCs) populations. Blocking the activation of STAT3 could reverse the effects^13, 17^. In line with this theory, we observed significantly decreased the population of MDSCs in peripheral blood and spleen. Collectively, current research has demonstrated that the activation of STAT3 boosted chemo-resistance, immune suppression and CSCs expansion. These effects translated into a pronounced delay of *in vivo* melanoma tumor growth which was, at least in part, dependent on intact immunity as evidenced by the restoration of tumor growth after CD4+ and CD8+ depletion. Thus, targeting STAT3 seems to be a favorable therapeutic tactic in the future clinical trial.

To sum up, our experiment and observation demonstrated the important role of STAT3 in the progression of ASCC, and the inhibition of STAT3 by S3I-201 led to delayed tumor growth and reversion of immunosuppression. STAT3 inhibitor has effects on both tumor and immunocytes that converge to mediate tumor rejection.

## Methods

### *Tgfbr1/Pten* 2cKO mice and ethics statement

The generation of *Tgfbr1/Pten* 2cKO (*K14*-*CreER* ^tam^; *Tgfbr1* ^flox/flox^; *Pten* ^flox/flox^) mice has been previously described^25^. The *Tgfbr1/Pten* 2cKO mice and their controls (*Tgfbr1* ^flox/flox^ *Pten* ^flox/flox^) were from the same litter. Mice were housed in specific-pathogen-free (SPF) conditions and all animal procedures were performed in compliance with the NIH guidelines for the Care and Use of Laboratory Animals and approved by the Animal Care and Use Committee of Wuhan University. One- to two-month-old mice received a tamoxifen induction procedure which has been previously described^11^.

### S3I-201 treatment

S3I-201 (NSC74859, S1155, Westlake Village, VA) was operation as previously described^26^. Two weeks after the last dose of tamoxifen, the mice were randomized into the control group (n = 10) or the experimental group (n = 10). The control group received PBS by intraperitoneal injection every other day (i.p. q.o.d). And the experimental group received 5 mg/kg S3I-201 by intraperitoneal injection every other day (i.p. q.o.d). Mice were treated with PBS or S3I-201 for 6 weeks (week 3 to week 9), and tumor size was measured weekly. Tumor volume was calculated by multiplying the three dimensions of each tumor using a digital micrometer caliper. Tumor burden was calculated as the individual tumor volume in each mouse and normalized with relative tumor growth by dividing the final volume by the initial tumor volume. At the end of the tumorigenesis studies determined according to a systematic evaluation by the veterinary doctor, the mice were euthanized; tissues were harvested and then fixed in paraffin overnight. Fixed tissues were then transferred to 70% alcohol and processed for paraffin embedding for a histopathological diagnosis and further studies. Some of the tissue was frozen at −80 °C for western blot analysis.

### Immunohistochemistry and immunofluorescence

Antibodies againstmouse Ki-67, p-STAT3, CD11b, Gr-1 were purchased from Abcam (Cambridge, UK). The sections of *Tgfbr1/Pten* 2cKO ASCC samples, as compared with *Tgfbr1/Pten* 2cKO anal skin, *Tgfbr1* ^*flox/flox*^ */Pten* ^*flox/flox*^ anal skin, and S3I-201 treated *Tgfbr1/Pten* 2cKO anal skin were stained with the antibodies by immunohistochemistry using an appropriate biotin-conjugated, secondary antibody and a MaxinSP kit (Vector Laboratories, Burlingame, CA). Slides were scanned using an Aperio ScanScope CS scanner (Vista, CA). Then the image data was processed with background subtraction and quantified using Aperio Quantification software (Version 9.1) for the membrane, nuclear, or pixel quantification. An area of interest was selected in either the epithelial or the cancerous area for scanning and quantification. Histoscore was calculated as previously described^27^. Briefly, four random high power field (20×)of each slide with membrane and nuclear immunostaining was calculated as a percentage of different positive cells using the formula: (1× the percentage of cells staining weakly positive) + (2× the percentage of cells staining moderately positive) + (3× the percentage of cells staining strongly positive)^28, 29^. Quantification of pixel intensity was calculated as total intensity/total cell number. The threshold for scanning of different positive cells was set by a pathologist according to the standard controls provided by Aperio.

For immunofluorescence, slides were hydrated in alcohol, washed three times in PBS, retrieved using sodium citrate in a pressure cooker, blocked with 2.5% bovine serum album in PBS buffer for 1 hour at 37 °C.Slides were then incubated with primary antibody overnight at 4 °C. The next day, slides were incubated with fluorochrome-conjugated secondary antibodies (Alexa 594 anti-mouse and Alexa 488 anti-mouse; Invitrogen, Carlsbad, USA) and mounted in Vectashield with DAPI (Vector Laboratories, CA, USA). Fluorescence images were captured using a fluorescence microscope.

### Flow cytometry analysis

FACS was performed on single cell suspensions from Spleen and blood in *Tgfbr1/Pten* 2cKO mice with or without STAT3 blockade. Single cell suspension from tumor tissues were acquired according to a protocol previously reported^30^. Briefly, tumor tissue was processed with a gentleMACS™ Dissociator and a murine tumor dissociation kit (Miltenyi Biotec, Bergisch Gladbach, Germany) to create a single cell suspension. Wild type controls with same dose tamoxifen were set for flow cytometry analysis. Single cell suspensions from spleen, blood and tumor were processed according to a standardized protocol. These cells were labeled with FITC-conjugated anti-mouse CD4, CD8, and CD11b (all from BD Biosciences, 554046, 553030, 561689); Percp-Cy5.5-conjugated anti-mouse F4/80 (from eBioscience,45–4801), PE-conjugated anti-mouse Gr-1 (from BD Bioscience, 562060); isotype-matched IgG controls (eBioscience, 12–4732). These cells were analyzed on a FACS caliber flow cytometer with CellQuest software and gated by the side scatter and forward scatter filters (Becton Dickinson, Mountain View, CA). Live cells were gated by 7AAD (Invitrogen) and populations phenotyped as described above.

### Western blot analysis

Harvested tissues were lysed in T-PER (Pierce, Rockford, IL) containing a complete mini-protease inhibitor cocktail and phosphate inhibitors (Roche, Branchburg, NJ). The skin around anal area was harvested from two individual *Tgfbr1* ^flox/flox^/*Pten* ^flox/flox^ mice and two Tgfbr1/Pten 2cKO mice;tumors harvested from *Tgfbr1/Pten* 2cKO mice were used for Western blot analysis. Antibodies against STAT3, p-STAT3 (T705), survivin were purchased from Cell Signaling Technology (Danvers, MA, USA). A total amount of 20 µg protein from each sample was denatured and then loaded on SDS-polyacrylamide gel. After the proteins were separated by electrophoresis, they were then transferred onto polyvinylidene fluoride membranes. Then the blot was detected by a chemiluminescence detection kit (West Pico, Thermo, MA, USA), β-actin was used as a control.

### Statistical analysis

Data analyses were done using Graph Pad Prism version 5.0 for Windows (Graph-Pad Software Inc., La Jolla, CA). One-way ANOVA followed by the post-Turkey or Dunnett multiple comparison tests were used to analyze the differences in immunostaining among each group or as compared with control group. The Mann-Whitney U test and Student t-testwere used to evaluate differences between the total tumor areas of the mice treated with S3I-201 as compared to untreated mice. A *P*-value under 0.05 was considered statistically significant (ns, *P* > 0.05; *P < 0.05; **P < 0.01; ***P < 0.001).

## Electronic supplementary material

Supplementary Information
