# Supplementary material for: Targeting phosphorylation of STAT3 delays tumor growth in HPV-negative anal squamous cell carcinoma mouse model
Source: Sci Rep. 2017 Jul 26;7:6629. doi: 10.1038/s41598-017-06643-9 (PMC5529522; doi:10.1038/s41598-017-06643-9)
Supplement: Supplementary file 1 — Supplementary Information [file 41598_2017_6643_MOESM1_ESM.pdf]

TITLE: Targeting phosphorylation of STAT3 delays tumor growth in HPV-negative anal squamous cell carcinoma mouse model

Lin-Lin Bu[a,b,1], Yi-Cun Li[a,1], Guang-Tao Yu[a], Jian-Feng Liu[a], Wei-Wei Deng[a], Wen-Feng Zhang[b], Lu Zhang [a,*], Zhi-Jun Sun[a,b,*]

a

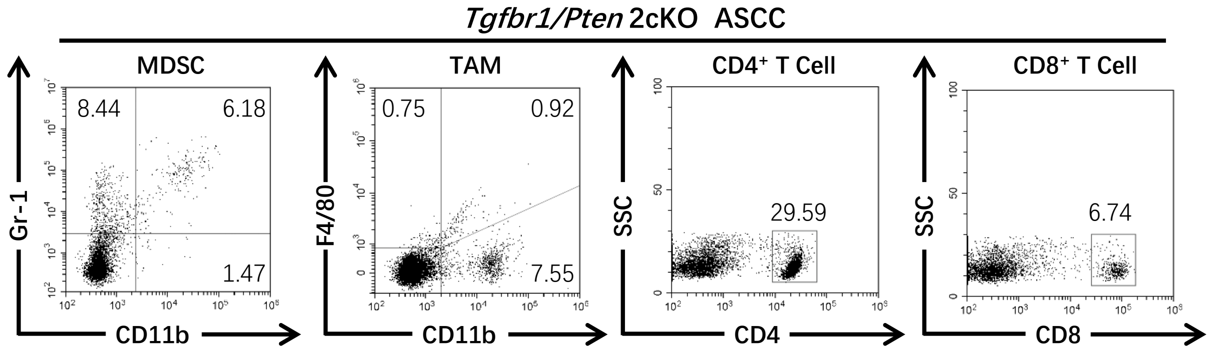

Supplementary Figure 1. Gating strategies of tumor infiltrating immune cells. (**a**) examples of the gating strategy of $CD11b^+Gr\text{-}1^+$, $CD11b^+$F4/80, $CD4^+$ and $CD8^+$ cells in the tumor bed.
